# Linear matrix genetic programming as a tool for data-driven black-box control-oriented modeling in conditions of limited access to training data

**DOI:** 10.1038/s41598-024-63419-8

**Published:** 2024-06-03

**Authors:** Tomasz Praczyk, Maciej Szymkowiak

**Affiliations:** Department of Computer Science, Polish Naval Academy, Gdynia, Poland

**Keywords:** Neuro-evolution, Modular neural networks, Recurrent neural networks, Genetic programming, Deep learning, Underwater vehicles, Computational science, Computer science, Information technology

## Abstract

In the paper, a new evolutionary technique called Linear Matrix Genetic Programming (LMGP) is proposed. It is a matrix extension of Linear Genetic Programming and its application is data-driven black-box control-oriented modeling in conditions of limited access to training data. In LMGP, the model is in the form of an evolutionarily-shaped program which is a sequence of matrix operations. Since the program has a hidden state, running it for a sequence of input data has a similar effect to using well-known recurrent neural networks such as Long Short-Term Memory (LSTM) or Gated Recurrent Unit (GRU). To verify the effectiveness of the LMGP, it was compared with different types of neural networks. The task of all the compared techniques was to reproduce the behavior of a nonlinear model of an underwater vehicle. The results of the comparative tests are reported in the paper and they show that the LMGP can quickly find an effective and very simple solution to the given problem. Moreover, a detailed comparison of models, generated by LMGP and LSTM/GRU, revealed that the former are up to four times more accurate than the latter in reproducing vehicle behavior.

## Introduction

Autonomous Mobile Robots (AMR) are the answer to the needs of the armed forces, logistics companies, agricultural industry, healthcare institutions, and warehouse systems in terms of improving efficiency, increasing speed, and ensuring precision and safety of operation.

AMRs are equipped with an Autonomous Control System (ACS) fed with a range of sensors and based mainly on artificial intelligence and machine learning algorithms. The ACS allows AMRs to move independently in the environment and perform assigned tasks without having to resort to human assistance. AMRs are most often able to independently plan a route, avoid collisions, and respond to emergencies.

However, when designing the ACS that would be able to perform all the tasks mentioned above, an AMR model is required at least in the initial phase. The ACS decision engines based on artificial intelligence algorithms require intensive training during which various engine variants are tested, and the implementation of this process based on real AMRs would be associated with its intensive use, enormous costs, as well as the aging of the mechanical components. Moreover, early versions of the ACS often have many bugs, which means that testing it on a real AMR could lead even to its loss.

On the other hand, designing the ACS using a model rather than an actual AMR may in turn lead to a situation where the system is unable to effectively control the robot. We may deal with this situation if the model is significantly different from the modeled robot. Consequently, the model should reproduce the behavior of the real AMR as closely as possible. This is especially important when very precise maneuvers from the AMR are required.

Although the model-based approach dominates the design of ACS, there are also model-free algorithms^1^. The most commonly used solution in the field of model-free control is the use of adaptive systems based on reinforcement learning (RL)^2–7^. In this case, the system learns to control an AMR online when interacting with the environment. Another model-free approach is data-driven control which requires prior offline learning with the use of previously collected input–output data and is based on the use of regression techniques^8^. However, despite their advantages, model-free control algorithms may have difficulties with quick convergence, as demonstrated in^3^.

In the case of the model-based approach, there are three different options, namely: white-box, gray-box, and black-box modeling. White-box modeling requires full knowledge of the structure of the system under consideration. It applies the laws of physics and its core is a set of differential equations that govern the behavior of the system. The most widely used mathematical model of AMR is the six-degree-of-freedom (DOF) motion model, the form of which depends on the design of the modeled robot. For example, for underwater AMRs, a commonly used model is the one proposed by Fossen^9–12^. On the one hand, the above model has many parameters that can be easily determined, for example: weight, geometry, buoyancy, number, and arrangement of the propellers. But, on the other hand, it also requires knowledge about hydrodynamic parameters (added mass, and linear and nonlinear damping coefficients) which makes the process of constructing a complete dynamical model of the vehicle at a sufficient accuracy very challenging or even impossible.

The solution to the above problem is the application of gray-box models, which assume some insight into the construction of the robot and the existence of model identification methods that estimate unknown parameters of the model. Depending on when the model identification is performed, the identification methods can be divided into offline and online methods. In the first case, the parameter estimation can be performed using data obtained from simulations^13–15^ or from measurements of the reaction forces when the robot is moving. The adjustment of the model parameters to the measurement data takes place with the use of the least squares algorithm^16,17^ or PSO^18^.

The disadvantage of offline methods is that the model parameters remain unchanged after the model optimization process is completed. Meanwhile, AMRs can change equipment and sensors depending on the mission they are performing. Each such change, in turn, changes the physical properties of the robot and, in consequence, also the model parameters determined earlier. The solution to this problem is the application of adaptive methods that estimate the unknown, uncertain, and nonlinear parameters online while the robot is moving. In this case, we are dealing with the whole range of adaptive strategies, such as: Support Vector Regression (SVR)^19^, Extended Kalman Filter^20,21^, neural networks^22–26^, neuro-fuzzy systems^27^ and fuzzy inference systems^28^.

A different modeling approach is presented by data-driven black-box methods that use only input and output data, which makes them independent of the robot design and thus applicable to various types of air, land, surface, and underwater robots. The structure of the model (linear or non-linear), which may be difficult to define due to the complexity and time variability of the system and its environment, is not necessary in this case. The dominant approach, in this case, is the use of neural networks, ranging from simple perceptron networks (shallow and deep)^29–33^, convolutional networks (CNN)^34,35^ to recurrent networks (RNN)^36–39^, mainly Long Short Term Memory (LSTM)^40–44^ and Gated Recurrent Unit (GRU)^45^ networks.

However, neural network modeling still encounters problems in determining the optimal values of network hyper-parameters such as, for example, the number of hidden layers and the number of nodes in each layer. In the case of networks containing LSTM/GRU units, which are now standard in time series forecasting and thus in the modeling of linear and non-linear dynamic systems, additional problems are the fixed architecture of LSTM/GRU units and data “hunger”. The units can only be organized in layers, there is no possibility of interfering with their internal architecture and thus adapting them to the problem to be solved. Moreover, LSTM/GRU networks as deep learning networks require numerous training datasets for training. When modeling dynamic systems, they require many sample recordings of system behavior. Unfortunately, in the case of real AMRs, multiple registrations of their behavior would be associated with huge costs, which means that the modeling of the robots must be based on data sets of limited size.

The solution to the above problems may be Linear Matrix Genetic Programming (LMGP), which is a matrix extension of Linear Genetic Programming (LGP)^46–48^. LMGP evolves LSTM/GRU-like networks, which take the form of linear programs, called Matrix Operation Programs (MOP), containing a sequence of matrix operations. MOPs evolve according to Hill Climb Modular Assembler Encoding (HCMAE)^49^ which is an algorithm meant for the evolution of matrices. In contrast to LSTM/GRU networks whose construction is strictly defined, the architecture of MOPs depends on the evolutionary process during which both simple and very complex models can be created.

To verify the effectiveness of the LMGP, it was applied to evolve a model of an autonomous underwater vehicle (AUV). The choice of an AUV as a modeling object and test field for LMGP was dictated by the fact that the construction of a reliable fast model of an AUV is required in the European Defence Agency Project No. B-746-ESM1-GP, entitled “Swarm of Biomimetic Underwater Vehicles”. It is necessary for the design of neural swarm-control ACE for AUVs whose purpose is to maintain various types of formations while moving in an environment with obstacles. This model is necessary because ACE training takes place in simulation conditions with the use of evolutionary techniques. The use of computationally demanding evolutionary techniques requires the use of an AUV model that is not only reliable but also fast enough so as not to slow down the training process too much.

In the project mentioned above, we are dealing with AUVs with a non-standard biomimetic drive (using fins as a drive). This makes the design of the model using traditional methods very difficult, and what is more, there is a risk that such a model will differ significantly from the modeling object. Moreover, the traditional model based on a system of differential equations may be very computationally complex and thus significantly slow down ACE training using population algorithms such as evolutionary algorithms. This means that a valuable alternative to traditional modeling methods may be data-driven methods that require only an example of the behavior of a real AUV. What is more, if they are implemented as neural network-like architectures they can significantly speed up calculations and training. Neural networks require an intensive and often long-lasting training process, but the trained networks in the use phase are much faster than traditional models based on differential equations.

The models designed during the research reported in the current paper were prepared using a small set of input-output training data recorded during the simulations, reported in^50^. This means that the task of LMGP, as well as rival methods, was to recreate another model specified in^51^. The use of a different model and not the actual AUV behavior to construct the data-driven model results from the fact that the actual target AUVs intended for cooperation in a swarm are not yet ready at the time of writing this paper. However, once these AUVs are ready, the method of constructing their model must be selected in advance, which results from the project schedule. Since the first tests using the LSTM and GRU networks as AUV data-driven models gave unsatisfactory results (the results of these networks are included in “Results”), it was decided to look for other solutions. The assumption is that these solutions must reliably recreate the behavior of a real object, must be fast enough so that when used together with evolutionary algorithms they do not slow down the ACS training process too much, and the process of training the models themselves must take into account the lack of access to large training data sets. This paper reports research aimed at answering the question of how to build AUV models that meet the above assumptions and whether the LMGP proposed in the paper can be the solution we are looking for in the project.

Various types of feed-forward and recurrent neural networks were used as reference points for LMGP, i.e. networks constructed with HCAE^52^, i.e. a light version of HCMAE intended for the evolution of small/medium-size monolithic neural networks, modular networks evolving with HCMAE, and networks with LSTM and GRU units. The latter networks did not evolve, they were trained with the use of classic gradient descent algorithms.

The contribution of the paper is as follows:

1. the LMGP is proposed which is a new technique for evolving data-driven black-box models in the form of LSTM/GRU-like recurrent neural networks,
2. the LMGP was experimentally verified by using it to construct a model of an AUV,
3. the LMGP was compared with different types of feed-forward and recurrent neural networks and training algorithms,
4. the models were built based on small sets of training data.

The rest of the paper is organized as follows: section two details LMGP, section three compares MOPs and LSTM/GRU networks, section four outlines HCMAE, section five reports the experiments, section six outlines limitations of the LMGP, section seven presents possible directions of further research, and the last eigth section contains the conclusions from the research.

Since many abbreviations are used in the paper, for the convenience of the reader, the list of the most frequently used abbreviations is presented in Table 1.

**Table 1:** List of abbreviations used throughout the paper.

Abbreviation	Definition
ACS	Autonomous control system
AMR	Autonomous mobile robot
AUV	Autonomous underwater vehicle
CNN	Convolutional neural networks
GRU	Gated recurrent unit
HCAE	Hill climb assembler encoding
HCMAE	Hill climb modular assembler encoding
LMGP	Linear matrix genetic programming
LSTM	Long short-term memory
MOP	Matrix operation program
RNN	Recurrent neural networks

## Linear matrix genetic programming

**Figure 1:**
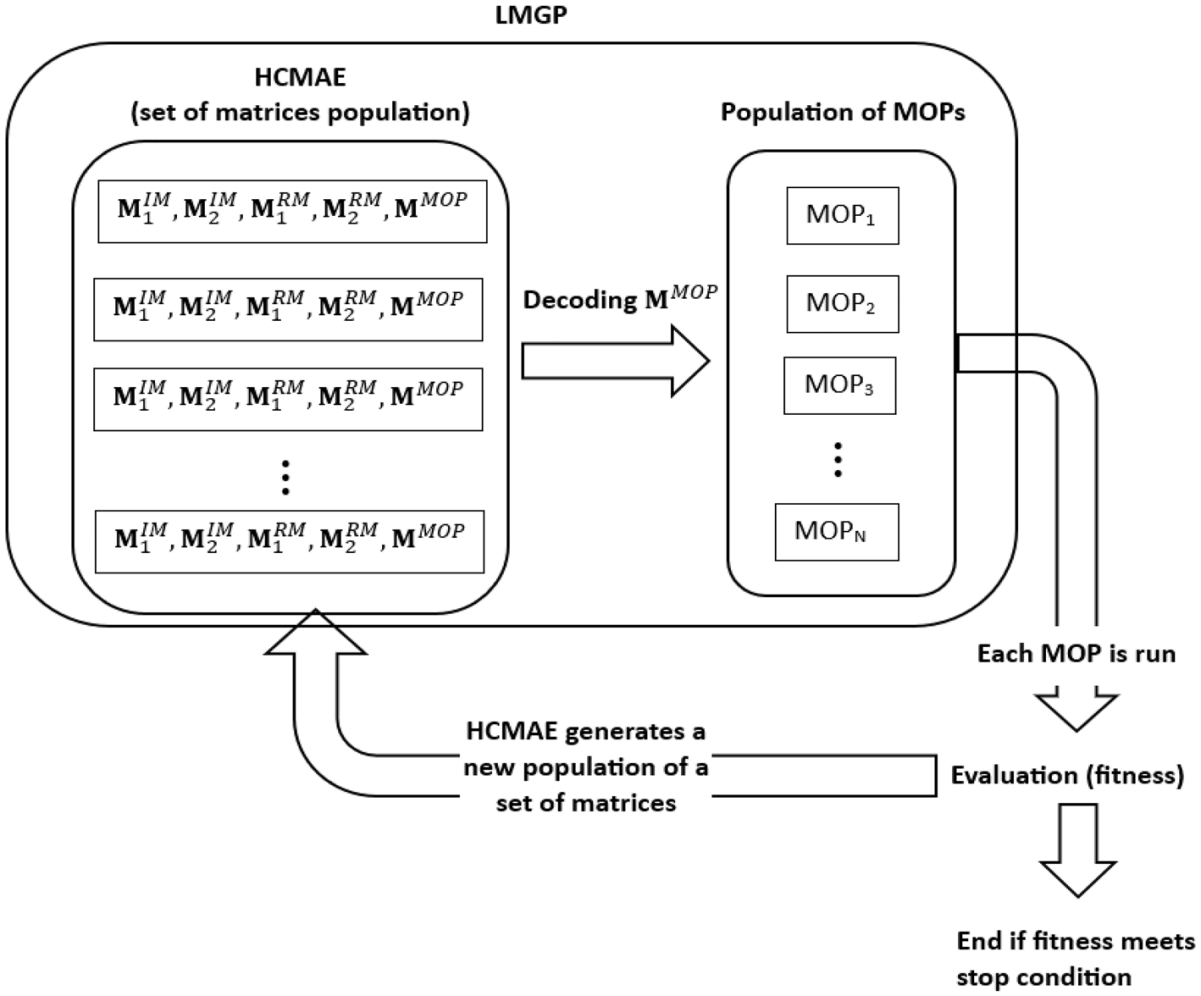
Scheme of LMGP for $N^{IM}=2$ and $N^{RM}=2$. In each iteration, HCMAE generates a population of matrix sets. Each set consists of $N^{IM}$ matrices ${\textbf{M}}^{IM}$, $N^{RM}$ matrices ${\textbf{M}}^{RM}$ and one matrix ${\textbf{M}}^{MOP}$. Each ${\textbf{M}}^{MOP}$ is decoded into MOP which is then evaluated. The evaluation of all MOPs is used by HCMAE to generated new matrix set.

The LMGP is an evolutionary technique meant for the construction of neural network data-driven black-box models represented in the form of Matrix Operation Programs (MOP). The MOPs evolve according to the HCMAE which is an algorithm for evolving a set of matrices and a LMGP optimization engine. The LMGP scheme is presented in Fig. 1.

The task of MOP is to predict the state of the modeled system based on a sequence of previous states and control signals. The current state of the system and the control signals are fed into the MOP input, and after executing the program, the predicted state is obtained at the MOP output.

MOP consists of a sequence of matrix operations: MOP$=<O_1,O_2, \ldots ,O_{N^O}>$, $N^O\le N^{O,max}$, where $N^{O,max}$ is maximum number of operations in all evolving MOPs. Execution of an MOP involves executing all operations in sequence, starting from operation $O_1$ and ending with operation $O_{N^O}$.

**Figure 2:**
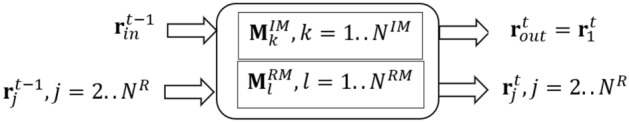
MOP operation over time. Like LSTM/GRU, MOP processes data sequentially and keeps its hidden state in registers ${\textbf{r}}_2, \ldots ,{\textbf{r}}_{N^R}$ through time (*t* - time step).

The task of each operation is to change the state of one of the MOP registers. The MOP operates on a set of registers: $<{\textbf{r}}_0={\textbf{r}}_{in},{\textbf{r}}_1={\textbf{r}}_{out},{\textbf{r}}_2, \ldots ,{\textbf{r}}_{N^R}>$, where ${\textbf{r}}_0 \in {\mathbb {R}}^{I^{length}}$, ${\textbf{r}}_1,\ldots ,{\textbf{r}}_{N^R} \in {\mathbb {R}}^{O^{length}}$, i.e. vectors that play the role of variables in the MOP. Each MOP has $N^R+1$ registers at its disposal, including an input register ${\textbf{r}}_{in}={\textbf{r}}_0$ and an output register ${\textbf{r}}_{out}={\textbf{r}}_1$. MOP operations $O_i$, $i=1\ldots {N^O}$ cannot change the state of the input register ${\textbf{r}}_{in}$. This register can only be modified externally by setting a new MOP input state. The operation of MOP over time is depicted in Fig. 2.

LMGP defines six main operations that were used in the experiments reported further:

1. Input Multiplication (IM): ${\textbf{r}}_i:={\textbf{M}}_j^{IM}{\textbf{r}}_{in}$, where $i=1..N^R$, $j=1..N^{IM}$, and ${\textbf{M}}_j^{IM}$ is a matrix of size $O^{length}\times I^{length}$,
2. Register Multiplication (RM): ${\textbf{r}}_i:={\textbf{M}}_j^{RM}{\textbf{r}}_k$, where $i,k=1..N^R$, $j=1..N^{RM}$, and ${\textbf{M}}_j^{RM}$ is a matrix of size $O^{length}\times O^{length}$,
3. Register Addition (RA): ${\textbf{r}}_i:={\textbf{r}}_j+{\textbf{r}}_k$, where $i,j,k=1..N^R$,
4. Register Addition and Element-Wise product (RAEW): ${\textbf{r}}_i:={\textbf{r}}_i+{\textbf{r}}_j \circ {\textbf{r}}_k$, where $i,j,k=1..N^R$,
5. Register Logistic (RL): ${\textbf{r}}_i:=logistic({\textbf{r}}_i)$, where $i=1..N^R$,
6. Register Hyperbolic Tangent (RHT): ${\textbf{r}}_i:=tanh({\textbf{r}}_i)$, where $i=1..N^R$.

However, the above list of operations is not exhaustive and, if necessary, it can be extended to include other operations, e.g.:

7. Bias Addition (BA): ${\textbf{r}}_i:={\textbf{r}}_i+{\textbf{M}}^B(k)$, where ${\textbf{M}}^B(k)$ is $k^{th}$ column of a bias matrix ${\textbf{M}}^B$ of size $O^{length}\times N^B$,
8. Register Addition Multiplication (RAM): ${\textbf{r}}_i:={\textbf{r}}_i + {\textbf{M}}_j^{RM}{\textbf{r}}_k$,
9. Register Element-Wise product (REW): ${\textbf{r}}_i:={\textbf{r}}_j \circ {\textbf{r}}_k$,
10. Register Negation (RN): ${\textbf{r}}_i:=-{\textbf{r}}_i$,
11. Register Constant Addition (RCA): ${\textbf{r}}_i:={\textbf{r}}_i + {\textbf{c}}$, where ${\textbf{c}}$ is constant vector.

The first operation in any MOP is always an IM. All other operations can be of any type.

**Figure 3:**
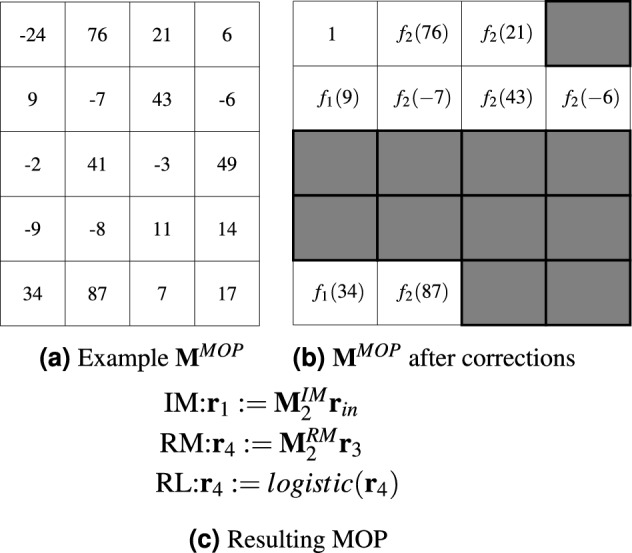
Example transformation of ${\textbf{M}}^{MOP}$ into MOP for $N^{O,max}=5$, $N^O=3$, $N^R=4$, $N^{IM}=2$, $N^{RM}=2$. The subsequent rows of ${\textbf{M}}^{MOP}$ encode the MOP operations in turn: ${\textbf{o}}_1^g=<-24,76,21,6>$, ${\textbf{o}}_2^g=<9,-7,43,-6>$, ${\textbf{o}}_3^g=<-2,41,-3,49>$, ${\textbf{o}}_4^g=<-9,-8,11,14>$, and ${\textbf{o}}_5^g=<34,87,7,17>$. Gray boxes correspond either to unused operation parameters or inactive operations due to $o^g_1 \le 0$. $O_1$ is always IM operation.

In addition to a set of registers-variables ${\textbf{r}}_i$, $i=0..{N^R}$, MOP also has at its disposal a set of matrices-constants ${\textbf{M}}_i^{IM}$, ${\textbf{M}}_j^{RM}$, $i=1..N^{IM}$, $j=1..N^{RM}$ whose state does not change while the program is running. Matrices ${\textbf{M}}_i^{IM}$ are used in IM operations while ${\textbf{M}}_j^{RM}$ are used in RM operations.

MOPs are constructed in an evolutionary manner using HCMAE. The HCMAE decides on the number of operations $N^O\le N^{O,max}$, the type of individual operations, the assignment of registers ${\textbf{r}}_i$, $i=2..{N^R}$ and matrices ${\textbf{M}}_i^{IM}$, ${\textbf{M}}_j^{RM}$, $i=1..N^{IM}$, $j=1..N^{RM}$ to individual operations, as well as the content of matrices ${\textbf{M}}_i^{IM}$ and ${\textbf{M}}_j^{RM}$.

At the genotypic level, each operation is encoded in the form of an integer vector ${\textbf{o}}^g=<o^g_1, \ldots ,o^g_4>\in {\mathbb {Z}}^4$, where $o^g_1$ determines type of operation, $o^g_2$ indicates left-hand side register, whereas $o^g_3$ and $o^g_4$ encode right-hand side matrices, registers, and constants. All operations of MOP are grouped in a matrix ${\textbf{M}}^{MOP}$ of size $N^{O,max} \times 4$ in which each row encodes a single operation—see Fig. 3.

To decode and create a MOP it is necessary to decode all operations. However, the final MOP only contains operations whose $o^g_1 \ge 1$. Rows of ${\textbf{M}}^{MOP}$ whose $o^g_1 \le 0$ correspond to inactive operations and they are not analyzed. This is the case except for the first row which always corresponds to the IM operation. The above mechanism enables LMGP to evolve variable-length MOPs.

To decode an operation, the operation type is decoded first, and then the other type-dependent components. The type of operation is decoded as follows: $$o^T:=f_1(o^g_1):=(o^g_1$$ **mod** $$N^T)+1$$, where $$o^T \in \{IM,RM,RA,RAEW,RL,RHT\}$$ is an operation type whereas $$N^T=6$$ is the number of operation types. In turn, registers and matrices used in the operation are decoded as follows: $$o^R_{2,3,4}:=f_2(o^g_{2,3,4}):=(abs(o^g_{2,3,4})$$ **mod** $$N^{max})+1$$, where $$o^R$$ is the register/matrix number and $$N^{max}$$, depending on the operation type and decoded component, is equal to $$N^R$$, $$N^{IM}$$ or $$N^{RM}$$. For example, if $$o^T=RM$$ then $${\textbf{r}}_i={\textbf{r}}_{o^R_2}$$ (left–hand register), $$N^{max}=N^R$$, $${\textbf{M}}_j^{RM}={\textbf{M}}_{o^R_3}^{RM}$$, $$N^{max}=N^{RM}$$, and $${\textbf{r}}_k={\textbf{r}}_{o^R_4}$$ (right–hand register), $$N^{max}=N^R$$.

## Comparing MOP to LSTM/GRU

**Figure 4:**
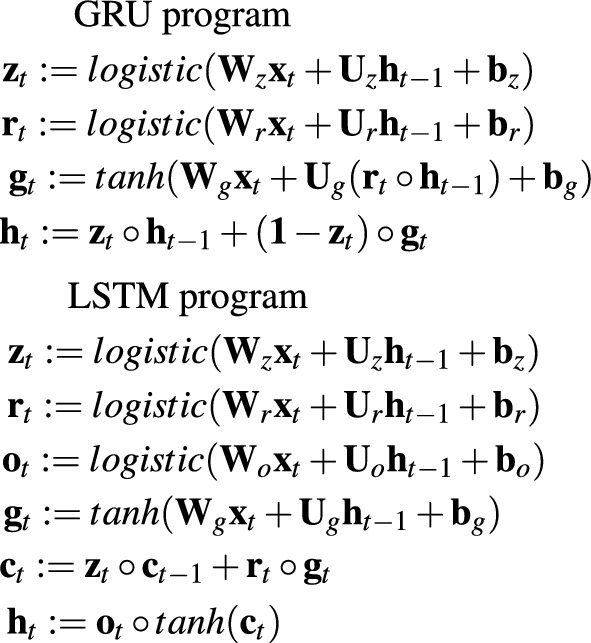
GRU and LSTM programs (*t* is point in time, ${\textbf{x}}_t \in {\mathbb {R}}^d$ is an input vector of size *d* (or input register like in MOP), ${\textbf{h}}_t \in <-1,1>^h$ is an output vector of GRU/LSTM layer (or output register line in MOP), ${\textbf{W}} \in {\mathbb {R}}^{h \times d}$, ${\textbf{U}} \in {\mathbb {R}}^{h \times h}$, ${\textbf{b}} \in {\mathbb {R}}^h$ are weight matrices and bias vectors, ${\textbf{z}}_t$, ${\textbf{r}}_t$, ${\textbf{o}}_t$ are different gate vectors (or hidden registers like in MOP), and ${\textbf{c}}_t$, ${\textbf{g}}_t$ are working vectors (or hidden registers like in MOP)).

As already mentioned, MOP is LSTM/GRU-like neural network organized in the form of a sequence of matrix operations. However, LSTM/GRU networks can also be represented as a sequence of matrix operations. Such representation of GRU and LSTM networks consisting of *h* units is depicted in Fig. 4.

**Figure 5:**
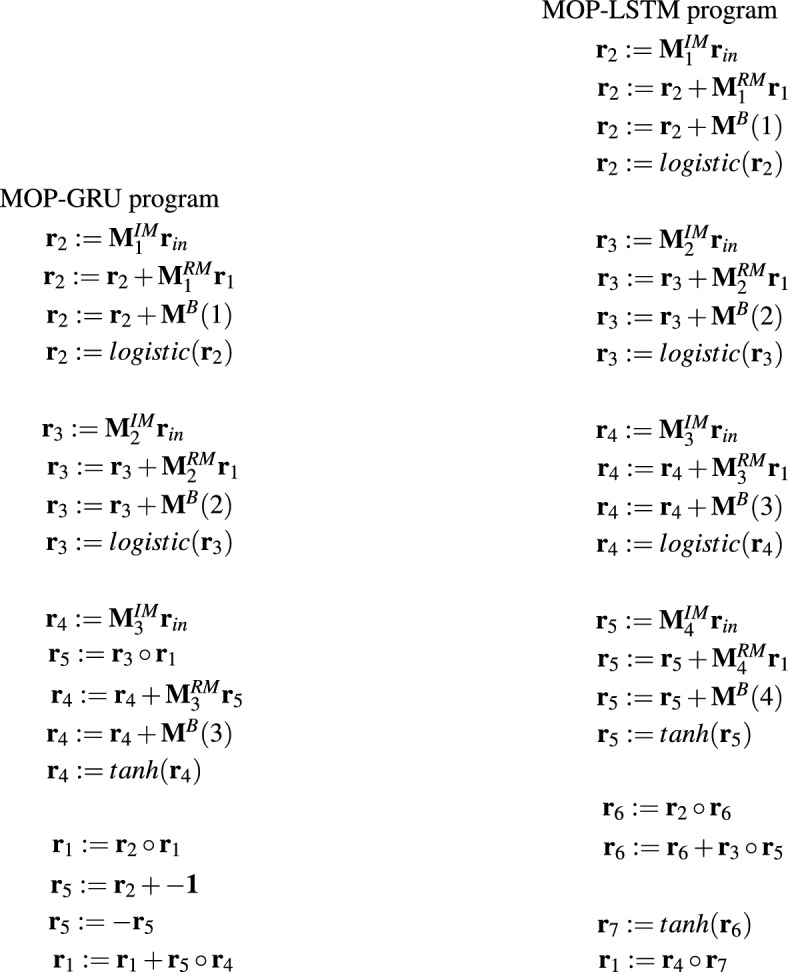
Example MOPs implementing GRU and LSTM networks—one block of operations corresponds to one operation in GRU/LSTM program (${\textbf{r}}_{in} \in {\mathbb {R}}^d$ is an input vector of size *d*, ${\textbf{r}}_1 \in <-1,1>^h$ is an output vector, ${\textbf{M}}^{IM} \in {\mathbb {R}}^{h \times d}$, ${\textbf{M}}^{RM} \in {\mathbb {R}}^{h \times h}$, ${\textbf{M}}^B(\cdot ) \in {\mathbb {R}}^h$ are weight matrices and bias vectors, ${\textbf{r}}_i$, where $i=1..5(7)$ are registers).

The operation of GRU/LSTM networks can also be implemented in the form of appropriate MOP programs. Figure 5 depicts MOP counterparts of GRU and LSTM programs specified in Fig. 4.

The above-mentioned MOPs and GRU/LSTM programs are equivalent in terms of the resources they use, i.e. registers/gates, weight matrices or bias vectors, and the output they generate. However, they differ in the way they operate. GRU/LSTM programs allow for greater parallelization of processing. The multiplication of the weight matrices with the input vector and gate vectors in each single operation (line in the program) can be done in parallel. Moreover, the calculation of the output of the individual gates is also mostly independent of each other and can be done in parallel.

Meanwhile, in MOP we deal with the sequential execution of a series of simple operations, each of which changes the state of one register. MOP counterparts of GRU/LSTM programs simply break down the GRU/LSTM functionality into smaller pieces that are executed sequentially.

However, while GRU/LSTM programs have a fixed architecture, and their operation can only be influenced by modifying the weight matrices and bias vectors, the advantage of MOPs is their flexibility which results from the fact that they can implement different behaviors not only by changing the content of *IM* and *RM* matrices, but also by adjusting the program code itself to the problem to be solved. MOPs can be very simple but also more complex like those presented in Fig. 5. The complexity of MOPs depends on the hyper-parameter setting, i.e. types of operations that are allowed in the programs, the maximum number of *IM* and *RM* matrices, the maximum number of registers, and decisions of the HCMAE optimization engine which designs the matrices and then assembles MOPs from ready-made parts.

## Hill climb modular assembler encoding

In order to construct a MOP, a program code and constants have to be defined, which in LMGP take the form of matrices. This means that the MOP optimization problem can be represented as a search for a set of optimal matrices. For this purpose, Hill Climb Modular Assembler Encoding (HCMAE) can be used, i.e. an algorithm for the evolution of modular neural networks in which each module is encoded in the form of a matrix. Since the algorithm is already detailed in^49^, in the current paper only its outline is given.

The HCMAE pseudo-code presenting a variant of the algorithm dedicated to the evolution of matrices (not neural networks) is shown in Algorithm 1. Generally, HCMAE represents an incremental approach meaning that it improves the matrices in many subsequent iterations. Initially, they are initiated as sparse matrices—(line 2 in Algorithm 1), and then, step by step, they are improved through gradually modifying their content (line 9 in Algorithm 1). In each iteration, only one matrix is modified, all other matrices remain unchanged. The selection of the matrix to update is random (line 8 in Algorithm 1). The probability of selecting a matrix, initially the same for all matrices (line 3 in Algorithm 1), changes in each HCMAE iteration and depends on the successes and failures in searching for its improved variant (line 18 in Algorithm 1). In each iteration, only matrix modifications leading to improved results are accepted (line 13 in Algorithm 1).

A key element of HCMAE is the way each of the selected matrices is modified to obtain improvement. Modification of the matrix in one iteration does not change its entire content, it usually concerns some fragment of the matrix. This means that we do not lose what was achieved earlier. The goal we want to achieve is to slightly change, add, or remove some matrix items, however, keeping all the positive changes obtained in the previous iterations.

Such an effect is achieved thanks to the evolutionarily shaped Assembler Encoding Program (AEP), which is a simple assembler-like program consisting of two/three operations and a sequence of data. The task of each operation is to transfer selected values from the AEP data sequence to the matrix to the place indicated by the operation parameters. The number of transferred values also depends on the parameters of the operation. Due to the small number of operations in AEP, matrix modifications usually have a small range. Moreover, the small size of the AEP enables its effective evolution.

**Algorithm 1:**
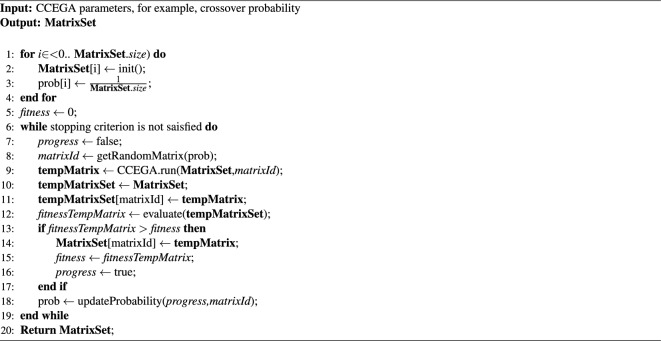
Pseudo–code of HCMAE.

In HCMAE, the evolution of AEPs, and consequently matrices, follows the Cooperative Co-Evolutionary Genetic Algorithm (CCEGA)^53,54^ (line 9 in Algorithm 1). Each operation as well as the data sequence evolve in separate populations. Operations are encoded in the form of integer strings (operation parameters indicating where to change the matrix, and what and how many values to copy), while the data sequences are encoded in the form of real-number strings.

In individual populations, evolution follows the classical genetic algorithm, with tournament selection, one-point crossover, and mutation. The probability of crossover is constant throughout the evolution, while the probability of mutation varies depending on the state of the evolutionary process. The operations and data sequences are crossed and mutated with different probabilities.

## Experiments

In order to verify the effectiveness of LMGP, the algorithm was applied to evolve a model of an AUV based on previously collected recordings of its behavior in an underwater environment with disturbances. The AUV carried out a mission by following the lawnmower trajectory a short distance from the bottom. Since the registration of the AUV behavior took place in simulation conditions, the task of LMGP was to recreate the mathematical model of the AUV used during simulations^50^.

### Modeling problem formulation

**Figure 6:**
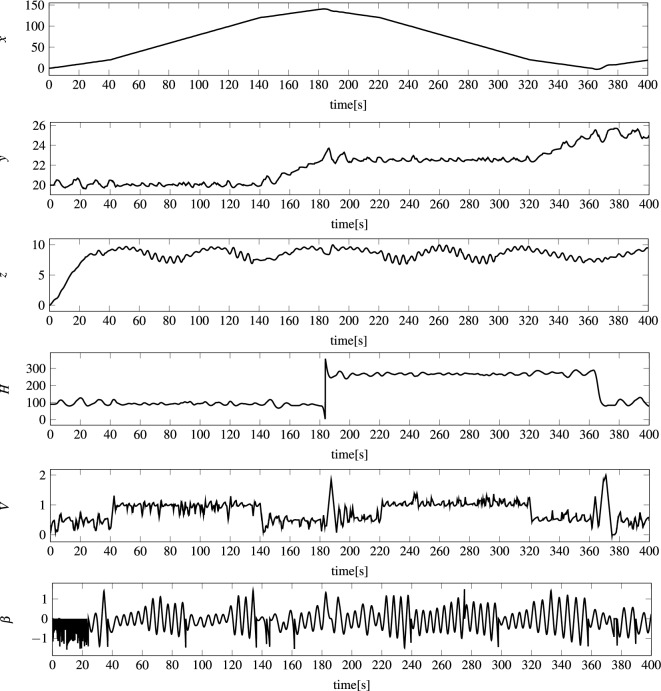
Example state of modeled AUV during 400 s voyage: (*x*, *y*, *z*) AUV position in a local coordinate system, *H* heading (direction), *V* speed, and $\beta$ pitch angle.

The state of the modeled vehicle ${\textbf{S}}=<x,y,z,H,V,\beta>$ (see Fig. 6) was determined by six parameters: (*x*, *y*, *z*)—AUV position in a local coordinate system, *H*—heading (direction), *V*—speed, and $\beta$—pitch angle. Moreover, the AUV was controlled by three control signals ${\textbf{C}}=<T,R,E>$: *T*—thrust of the propeller (forward), *R*—rudder angle (left/right), and *E*—elevator angle (down/up). This means that the task of the model was to predict the state of the vehicle ${\textbf{S}}(t+1)$ for the known current state of the vehicle ${\textbf{S}}(t)$, the previous states, the control signal ${\textbf{C}}(t)$, and the previous control signals.

**Figure 7:**
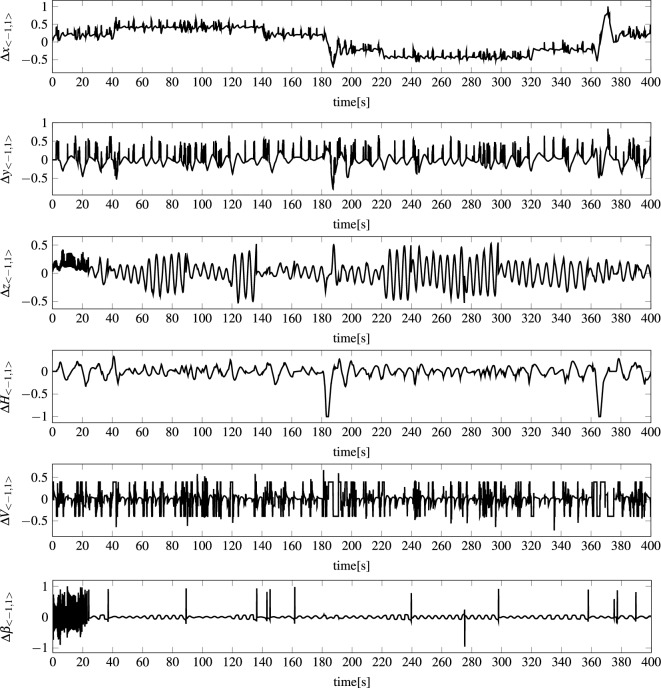
Example normalized state of modeled AUV during 400 second voyage. Each normalized state parameter was calculated according to the following procedure: $\Delta {\textbf{S}}_{<-1,1>}(t+1):=N_{<-1,1>}(\Delta {\textbf{S}}(t+1))$, $\Delta {\textbf{S}}(t+1):={\textbf{S}}(t+1)-{\textbf{S}}(t)$, where $N_{<-1,1>}$ is a normalization function.

**Figure 8:**
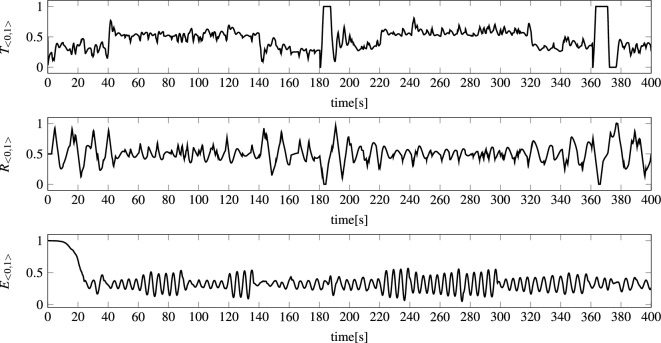
Example normalized control signals of modeled AUV during 400-s voyage. Each normalized control signal was calculated according to the following procedure: ${\textbf{C}}_{<0,1>}(t+1):=N_{<0,1>}({\textbf{C}}(t+1))$, where $N_{<0,1>}$ is normalization function.

For the feed-forward networks to be able to perform the above task, it was slightly reconfigured. Namely, the networks were not fed with combined vectors of the state and control signals: ${\textbf{S}}(t) \Vert {\textbf{C}}(t)$, but with the change of the state in two consecutive time steps concatenated with the control signals: $\Delta {\textbf{S}}_{<-1,1>}(t+1) \Vert {\textbf{C}}_{<0,1>}(t+1)$, (see Figs. 7 and 8) where $\Delta {\textbf{S}}_{<-1,1>}(t+1):=N_{<-1,1>}(\Delta {\textbf{S}}(t+1))$, $\Delta {\textbf{S}}(t+1):={\textbf{S}}(t+1)-{\textbf{S}}(t)$, ${\textbf{C}}_{<0,1>}(t+1):=N_{<0,1>}({\textbf{C}}(t+1))$, whereas $N_{<-1,1>}$ and $N_{<0,1>}$ are normalization functions.

Moreover, the task of the networks was not to predict the following state ${\textbf{S}}(t+2)$ but the normalized difference between this state and the current state: $\Delta {\textbf{S}}_{<-1,1>}(t+2):=N_{<-1,1>}({\textbf{S}}(t+2)-{\textbf{S}}(t+1))$. Finally, the predicted state was calculated as follows: ${\textbf{S}}^P(t+2):={\textbf{S}}(t+1)+{1/N}_{<-1,1>}(\Delta {\textbf{S}}_{<-1,1>}(t+2))$, where ${1/N}_{<-1,1>}$ is a reverse normalization function.

### Neural networks

In the experiments reported further, LMGP and MOP models were compared with:

1. Monolithic feed-forward (FFANN) and recurrent neural networks (RANN) evolving according to HCAE,
2. Modular feed-forward (MFFANN) and recurrent neural networks (MRANN) evolving according to HCMAE,
3. LSTM networks trained with Stochastic Gradient Decent algorithm,
4. GRU networks trained with Stochastic Gradient Decent algorithm.

The list of all neural networks and types of MOPs applied in the experiments is given in Tables 2 and 3

**Table 2:** List of neural networks used in the experiments (*FF* feed-forward, *R* recurrent)..

	FF/R	Max number of neurons	Number of modules	Training
FFANN	FF	30	1	HCAE
RANN	R	30	1	HCAE
MFFANN1	FF	40	2	HCMAE
MRANN1	R	40	2	HCMAE
MFFANN2	FF	40	3	HCMAE
MRANN2	R	40	3	HCMAE
MFFANN3	FF	40	3 + KMeans module	HCMAE
MRANN3	R	40	3 + KMeans module	HCMAE
LSTM1	R	6 LSTM units	1 layer	Gradient
GRU1	R	6 GRU units	1 layer	Gradient
LSTM2	R	12 LSTM units	2 layers	Gradient
GRU2	R	12 GRU units	2 layers	Gradient

**Table 3:** List of MOPs used in the experiments. .

	$N^{O,max}$	$N^{IM}+N^{RM}$	$N^{R}$	Number of MOP modules	Number of HCMAE matrices
MOP1	9	4	3	1	4
MOP2	9	8	3	1	2
MOP3	9	8	5	1	2
MOP4	9	8	3	1	2
MOP5	10	8	6	2	3
MOP6	10	8	6	2	3

**Figure 9:**
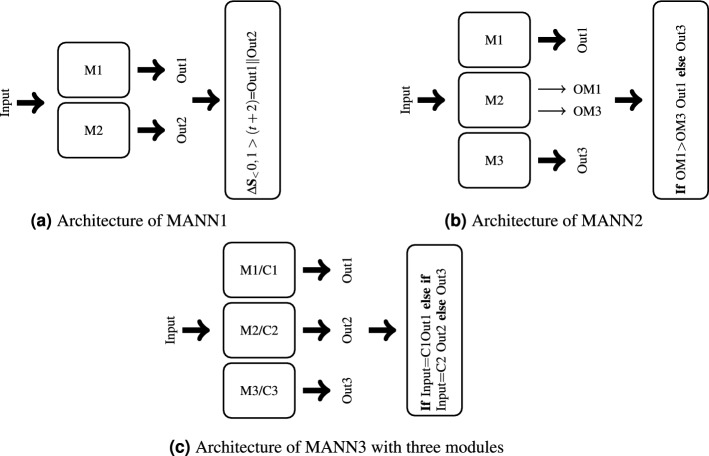
Architecture of MANN1, MANN2, and MANN3 (Input=$\Delta {\textbf{S}}_{<-1,1>}(t+1) \Vert {\textbf{C}}_{<0,1>}(t+1)$, in MANN1: Out1 = $\Delta<x,y,z>_{<-1,1>}$, Out2 = $\Delta<H,V,\beta>_{<-1,1>}$, in MANN2 and MANN3: Out*i* = $\Delta {\textbf{S}}_{<-1,1>}^{Mi}(t+2)$ where *i* is the number of module).

Monolithic networks have a loose, non-layered architecture and contain neurons with the logistic sigmoid activation function. In feed-forward networks, all but recurrent connections are allowed, while in recurrent networks there are no restrictions on the inter-neural connections. In each evolved network, the number of neurons and connections is determined evolutionarily. However, the total number of neurons, including input and output neurons, cannot exceed the maximum value which in the experiments amounted to 30: 9 input, 6 output, and a maximum of 15 hidden neurons.

Modular networks had three different architectures depicted in Fig. 9. In all the networks, regardless of the architecture, the maximum number of neurons in each module amounted to 20: 9 input, 6 output, and a maximum of 5 hidden neurons. Like in the monolithic networks, the exact number of neurons and connections depended on the evolutionary process. The inner architecture of the modules was the same as the architecture of monolithic networks.

The architecture MANN1 (see Fig. 9a) had two variants, i.e. feed-forward (FFMANN1) and recurrent (RMANN1). It consisted of two separate network-modules each fed with the entire input vector $\Delta {\textbf{S}}_{<-1,1>}(t+1) \Vert {\textbf{C}}_{<0,1>}(t+1)$. In FFMANN1 both modules were feed-forward, whereas in RMANN1 both modules were recurrent. The task of module M1 was to predict half of the AUV next state vector, while the task of module M2 was to predict the other half of this vector.

The MANN2 architecture consisted of three modules—see Fig. 9b. Like the MANN1, the MANN2 also had feed-forward (FFMANN2) and recurrent (RMANN2) variants. In FFMANN2, all the modules were feed-forward, whereas in RMANN2, modules M1, and M3 were recurrent and M2 was feed-forward. The task of modules M1, and M3 was to predict the next state of AUV, whereas M2 was responsible for selecting one of the predictive modules.

The MANN3 architecture was a combination of neural networks and the KMeans clustering algorithm. The input space of the modeled system (vector state plus control signals) was clustered into $N^C=2,3$ or 4 groups, each represented by a center (C1..C4) and a module (M1..M4). To predict the state of AUV at time-point $t + 2$, the input vector corresponding to time-point $t+1$ was assigned to one of the groups. The module associated with this group then made predictions. In the feed-forward variant (FFMANN3), all the modules were feed-forward, whereas in the recurrent variant (RMANN3) all the modules were recurrent.

The LSTM and GRU networks had one or two hidden layers (LSTM1/GRU1: one-layered networks, LSTM2/GRU2: two-layered networks), each layer with six LSTM/GRU units.

All the networks specified above were compared to six different MOPs, say, MOP1, MOP2,..., and MOP6. MOP1 had the following parameters: $N^{O,max}=9$, $N^R=3$, $N^{IM}=2$, $N^{RM}=2$, $I^{length}=9$, $O^{length}=6$, and during evolution, it was encoded in the form of five separate matrices, i.e. one ${\textbf{M}}^{MOP}$ matrix, two ${\textbf{M}}^{IM}$ matrices and two ${\textbf{M}}^{RM}$ matrices.

MOP2 was an extension of MOP1 with two further ${\textbf{M}}^{IM}$ matrices and two ${\textbf{M}}^{RM}$ matrices ($N^{IM}=4$, $N^{RM}=4$). Moreover, MOP2 was encoded as two separate matrices, i.e. one ${\textbf{M}}^{MOP}$ matrix and one super-matrix of size 4$O^{length}\times (I^{length}+O^{length})$ that included four ${\textbf{M}}^{IM}$ matrices and four ${\textbf{M}}^{RM}$ matrices. This procedure aimed to reduce the number of matrices processed by HCMAE. In MOP2, instead of nine separate matrices, HCMAE dealt with only two matrices. Another reason for the evolution of MOP2 as two separate matrices is the limitation of the current GPU HCMAE implementation which does not allow the evolution of such many matrices as in MOP2.

MOP3 extended MOP2 with two further registers ($N^R=5$). The evolution of MOP3 took place in two separate matrices like in MOP2.

MOP4 was MOP2 with a slight change in the process of matrices ${\textbf{M}}^{MOP}$ evolution. Normally, HCMAE treats all matrices the same way. It does not pay attention to the fact that they represent different entities, i.e. either a list of MOP operations or MOP matrices. In HCMAE, each genotype represents a change in a selected matrix. The change is made on selected items in the matrix—the items can be updated or set to zero. Which items are modified does not depend on what information these items store. The only adjustable parameter that can be set for each matrix is the range of changes. Usually, larger matrices need greater ranges than smaller matrices.

In MOP4, the other approach was applied, namely, ${\textbf{M}}^{MOP}$s evolved differently from the remaining matrices. In this case, each HCMAE genotype had three options, i.e.: (i) to remove the last operation from MOP, (ii) to modify the last operation in MOP, or (iii) to add a new operation at the end of MOP. Parameters for a new/modified operation were taken from the genotype.

MOP5 and MOP6 were modular programs which consisted of two MOP modules. In MOP5, the MOP modules were organized like modules M1 and M2 in MANN1 (see Fig. 9a), that is, each module was responsible for predicting half of the AUV state vector. In turn, MOP6 had a two-layered architecture like LSTM2/GRU2 networks, that is, the first layer MOP module produced input for the second layer MOP module.

Regardless of the modular option, all MOP modules had similar architecture: $N^{O,max}=5$, $N^R=3$, $N^{IM}=2$, $N^{RM}=2$. They differed in $I^{length}$ and $O^{length}$. In MOP5, both modules had the same input and output size: $I^{length}=9$ and $O^{length}=3$, whereas in MOP6, the input module was larger than the output module: $I^{length}=9$ and $O^{length}=6$ for the input module and $I^{length}=6$ and $O^{length}=6$ for the output module.

MOP5 and MOP6 evolved in three separate matrices, i.e. two ${\textbf{M}}^{MOP}$ matrices—one matrix per module, and one super-matrix including ${\textbf{M}}^{IM}$ and ${\textbf{M}}^{RM}$ sub-matrices—four sub-matrices per module.

### Dataset

In the experiments, 40 recordings of the modeled AUV were used. The behavior of the AUV was recorded at a frequency of 10 Hz. Each recording corresponded to a 400-s voyage. 20 recordings were used to evolve/train the models and the other 20 recordings were applied to verify their effectiveness.

From the point of view of the high demand of deep learning algorithms for training data, the number of 20, 400-s recordings intended for training the networks seems very small. However, such a number of data is a deliberate choice resulting from the desire to recreate the real situation in which, due to the high costs of recordings at sea, the number of available training data is limited.

### Evaluation

To evaluate models evolved according to LMGP, HCAE, and HCMAE, the following fitness function was used:

1 $$\begin{aligned} F_{\text {training}}(\text {model})= & {} \frac{1}{1+E_{\text {sum}}} \end{aligned}$$

2 $$\begin{aligned} E_{\text {sum}}= & {} \sum _{l=1}^{N_{20}}\sum _{t=0}^{N_{400}}E_{\text {entry}}(l,t) \end{aligned}$$

3 $$\begin{aligned} E_{\text {entry}}(l,t)= & {} {\left\{ \begin{array}{ll} 10|{\textbf{S}}| &{} Out_{l,t,k}=0.5 \; \forall k=1..|{\textbf{S}}|\\ \sum _{k=1}^{|{\textbf{S}}|}(E(l,t,k))^2 &{} \text {otherwise} \end{array}\right. } \end{aligned}$$

4 $$\begin{aligned} E(l,t,k)= {\textbf{S}}_{k,l}(t+2)-({\textbf{S}}_{k,l}(t+1)+Out_{l,t,k}) \end{aligned}$$

where

- $Out_{l,t,k}$—is *k*th output of the model in *l*th recording and *t*th recording entry,
- $N_{400}$—is the number of entries in a single recording, $N_{400}=3998$,
- $N_{20}$—is the number of recordings used in the training process, $N_{20}=20$.

The function (1) is the inverse of error $E_{\text {sum}}$ that is the sum of errors $E_{\text {entry}}$ determined for all recordings used in the training process and all entries in these recordings. In turn, error $E_{\text {entry}}$ is either the total error of reproducing all parameters of the modeled object $\sum _{k=1}^{|{\textbf{S}}|}(E(l,t,k))^2$ or penalty $10|{\textbf{S}}|$ when the model is a local maximum which generates a value of 0.5 on each of its outputs $Out_k$, $k= 1..|{\textbf{S}}|$, regardless of the modeled object’s state

In the case of LSTM/GRU networks, the standard MSE loss function was used in the network training process.

The final evaluation of the models was performed using the following function:

5 $$\begin{aligned} F_{\text {final}}(\text {model})= & {} \frac{1}{1+dist(E_{{\textbf{0}}},E_{\text {max}})} \end{aligned}$$

6 $$\begin{aligned} dist(E_{{\textbf{0}}},E_{\text {max}})= & {} \sqrt{\sum _{k=1}^{|{\textbf{S}}|}\left( E_{\text {max}}(k)\right) ^2} \end{aligned}$$

7 $$\begin{aligned} E_{\text {max}}(k)= & {} \max \limits _{l=21..40,t=0..N_{400}}(|E(l,t,k)|) \end{aligned}$$

where $l=21..40$ means the evaluation on the twenty recordings that were not used in the training process. Function (5) corresponds to a scalarization of a multi-objective optimization problem in which the goal is to minimize the Euclidean distance $dist(E_{{\textbf{0}}},E_{\text {max}})$ between maximum error point $E_{\text {max}}=<E_{\text {max}}(1), E_{\text {max}}(2), \ldots ,E_{\text {max}}(|{\textbf{S}}|)>$, which represents the combination of the largest errors for all tested cases and all vehicle parameters, and ideal point $E_{{\textbf{0}}}=<0,0,\ldots ,0>$, which indicates the perfect situation when all errors are equal to zero for each *k*, *l*, and *t*.

### HCMAE setting

In general, HCMAE parameters remained largely unchanged compared to the research reported in^49^. The only parameters that were tuned to the modeling problem were ranges of matrix modifications (number of updated items) by individual AEP operations. For matrices ${\textbf{M}}^{IM}$ and ${\textbf{M}}^{RM}$, the maximum and minimum ranges amounted to 50 and 5, respectively, while for matrix ${\textbf{M}}^{MOP}$ they amounted to 5 and 1. The remaining HCMAE parameters are given in the last section of the paper.

### Implementation

During the research, our own parallel (GPU, CUDA-NVIDIA parallel computing platform) implementation of LMGP in C++ was used. Both the optimization/evolutionary engine HCMA/HCMAE and the neural networks FFANN, RANN, MFFANN, MRANN, and MOPs had their parallel implementation in C++.

In turn, the LSTM/GRU networks as well as the process of their learning and testing were implemented in Python on GPU servers using the Keras library.

### Results

**Figure 10:**
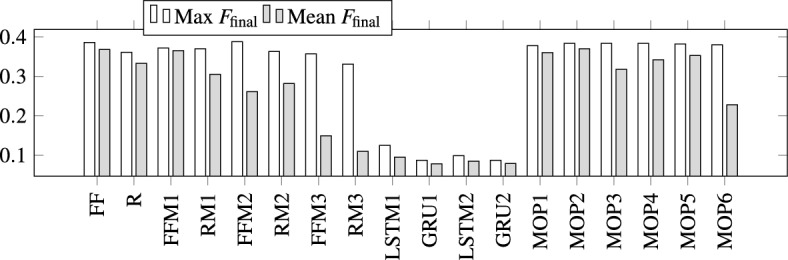
Results of experiments.

In order to compare all the modeling methods, each of them was used to generate thirty different models, each of which represented one run of the training process. Each run delegated a single most effective model with the highest evaluation according to function (1) or MSE loss function. The final evaluation in the form of mean and maximum values of the function (5) are given in Table 4 and Fig. 10.

**Table 4:** Results of experiments (the best results are bolded, FF means FFANN, R means RANN, and similarly for other neural networks).

	FF	R	FFM1	RM1	FFM2	RM2	FFM3	RM3	LSTM1
Max $F_{\text {final}}$	0.386	0.361	0.372	0.37	**0.388**	0.363	0.357	0.331	0.125
Mean $F_{\text {final}}$	0.368	0.333	0.365	0.305	0.261	0.282	0.149	0.11	0.095
Std. $F_{\text {final}}$	0.019	0.029	0.002	0.102	0.12	0.08	0.129	0.133	0.012

	GRU1	LSTM2	GRU2	MOP1	MOP2	MOP3	MOP4	MOP5	MOP6
Max $F_{\text {final}}$	0.087	0.099	0.087	0.378	0.384	0.384	0.384	0.382	0.38
Mean $F_{\text {final}}$	0.078	0.085	0.079	0.36	**0.37**	0.318	0.342	0.353	0.228
Std. $F_{\text {final}}$	0.003	0.004	0.004	0.025	0.013	0.094	0.08	0.057	0.107

The maximum values in the table and the figure show the result of the best model that was generated for a given modeling method out of thirty available models after the learning process. It turns out that LMGP, regardless of the MOP type, is the most effective in this respect. All MOPs except MOP1 achieved a fitness greater than 0.38. Only evolutionary neural networks FFANN and FFMANN2 achieved similar effectiveness.

However, if, when evaluating the methods, we take into account the average result, which shows the true potential of a given modeling method to generate effective models, then the most effective methods are LMGP(MOP1, MOP2, MOP5), HCAE(FFANN) and HCMAE(FFMANN1). Each of the above methods resulted in an average fitness above 0.35.

**Figure 11:**
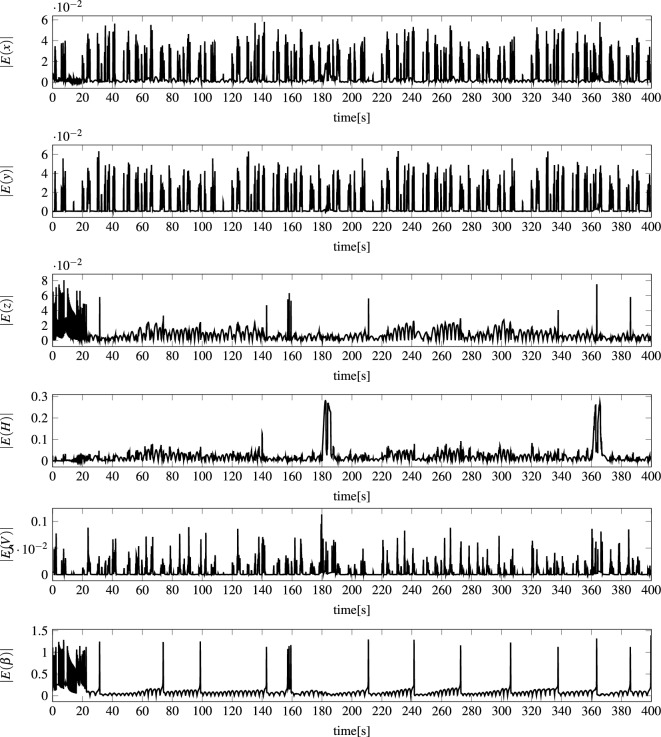
Example vehicle state prediction errors |*E*(*l*, *t*, *k*)| for MOP1 and for $t=1..N_{400}$, $k=1..|{\textbf{S}}|$—absolute errors for an example 400 second voyage for all vehicle state parameters.

Analyzing the state prediction errors generated by the most effective models mentioned above, examples of absolute errors are presented in Fig. 11, while examples of actual values are shown in Fig. 6, it should be stated that errors of position (*x*, *y*, *z*) are at a very low level. This is probably due to the fact that the position of the vehicle, regardless of the coordinate, is slowly changing. The error of heading *H* is also very small with two major spikes when the heading crosses 360 degrees and there is a drastic spike in value. The error of vehicle speed *V* is also at a small satisfactory level compared to the real values. The biggest errors appear for the pitch angle $\beta$. The reason is the large spikes in the input values of the models for this angle, which can be seen in Fig. 7. During the tests, all models were not supplied with the vehicle state parameters but with the change of the state in two consecutive time steps normalized to the range $<-1.1>$–$\Delta {\textbf{S}}_{<-1,1>}$. As Fig. 7 shows, the largest spikes in the input values to the models occur precisely for the pitch angle.

Noteworthy are the extremely poor results of the LSTM and GRU networks. Surprisingly, during the simulations, it was not possible to obtain even a single LSTM/GRU network that would be as effective as other modeling techniques. There are two most likely reasons for this state of affairs. First, the training dataset may not have been enough to make the networks more efficient. Second, they are the only networks that have not been trained evolutionarily. The use of the gradient training algorithm in their case could cause the objective function to get stuck in the local minima, which in turn could affect the mean value of the error.

Due to the population nature of the HCAE and HCMAE evolutionary algorithms, the learning process of MOPs and FFANN, RANN, MFFANN, MRANN neural networks was slower than the LSTM/GRU networks trained with the use of gradient algorithms in which a single instance of the network is trained. Regardless of the algorithm, the learning process ended each time when no progress was found for a longer period.

However, learning speed is not a key parameter in the construction of a reliable model of any real object. The most important in this case are two parameters. The first is the quality of the model, which is presented in Table 4 and Fig. 10 and is understood as its accuracy in reproducing the behavior of the real object. The second parameter is the speed of the model, which is important, for example, during its use in the process of building/learning the ACS system for the modeled object. The speed of the models is influenced by the implementation which was different for LSTM/GRU and the other models, but also by the “size” of each model and the potential of each of them for parallel processing and optimal use of GPU resources. In this case, it should be noted that although during the tests the differences between the various models were barely noticeable, the MOPs, due to their sequential nature, will always be slightly slower than other models of similar size.

Although MOPs are not significantly more effective than evolutionary neural networks, they have one feature that can make them a more attractive modeling tool. Namely, they constitute a comprehensible representation of the modeled object. Neural networks are a classic black-box model. The complex internal architecture of the networks makes it difficult or even impossible to analyze the modeled object based on the analysis of the networks. In the case of simple networks consisting of a few neurons, it is possible to analyze the impact of individual interneuron connections and the neurons themselves on the state of the modeled object. It is similar to larger networks with clearly separated building blocks, in which analysis is possible at the level of blocks, not neurons or connections. However, for more complex networks, for example, those constructed using HCAE/HCMAE which are monolithic or modular networks with monolithic modules without clearly separated layers or blocks, the analysis of their operation comes down to the level of a single neuron and synapse, which for people unfamiliar with neural networks seems to be impossible.

Unlike the networks, MOPs offer this possibility. Representing a modeled object in the form of a MOP, which is a sequence of well-known matrix operations, makes the object easier to understand. In the case considered in this paper, the neural models are encoded in the form of a matrix in which each entry indicates the weight of a connection between neurons. In addition, the matrix also includes neuron parameters such as bias or type of transfer function. Analysis of such a model, even for medium-size networks that were used in the experiments, is at least problematic. Meanwhile, most effective MOPs, regardless of their type, consisted of a single IM operation, which generated the output of the entire program—the result of the operation was saved in register no. 1 which in all MOPs played the role of an output register: ${\textbf{r}}_1:={\textbf{M}}^{IM}{\textbf{r}}_{in}$. Such MOPs revealed that the behavior of the modeled vehicle can be approximated with a high accuracy by a simple linear system and complex neural networks were unnecessary in this case.

**Figure 12:**
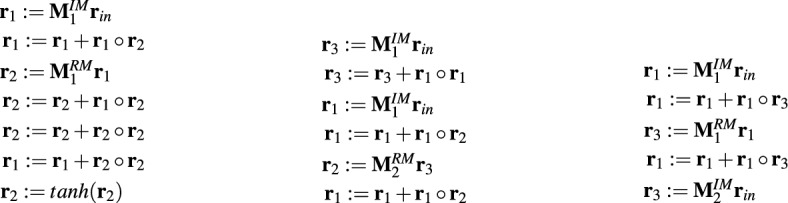
Example effective MOP models consisting of more than one operation.

In addition to the simple MOP models mentioned above, LMGP also evolved more complex models which were equally accurate as the simple ones. Examples of such models are given in Fig. 12. Despite their greater complexity, these models still seem simpler to analyze than their neural black-box counterparts.

The examples of MOPs depicted in Fig. 12 also show that although each of them is a sequence of operations, they enable the implementation of multiple parallel processing streams, each of which is associated with a different register. The more registers and matrices from which the MOPs can be assembled, the greater the possibility of building more complex models. However, increasing the number of potential MOP components increases simultaneously the complexity of the task of combining these components into a single model and makes it difficult to construct effective models. This is visible in the case of MOP3 which is an option with the largest number of available registers and matrices.

**Figure 13:**
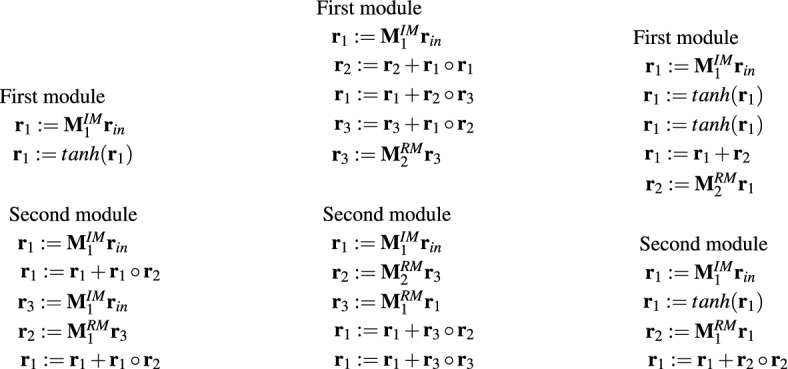
Example effective MOP5 models.

The experiments also revealed the key influence of the slow gradual evolution of matrices ${\textbf{M}}^{MOP}$, that encode list of MOP operations, on LMGP effectiveness. Faster and deeper changes of these matrices in subsequent iterations of the evolutionary process significantly hinder the generation of effective programs, as demonstrated by preliminary experiments with LMGP. For that reason, in all LMGP options, genetic operations on ${\textbf{M}}^{MOP}$ are very delicate and they are made on small fragments of the matrices. Most options simply modify some vertical or horizontal patch in the matrix whereas MOP4 adds or removes one operation at the end of the program.

In contrast to single-program MOPs, their modular counterparts MOP5 and MOP6 very rarely implemented simple linear models. In MOP5, which like MANN1 implemented two parallel streams of processing and which like MANN1 appeared to be an effective modeling solution, although each module was responsible for a piece of the entire task, there was not even a single linear module. What is more, a lot of modules consisted of the maximum number of operations. Example MOP5s are depicted in Fig. 13.

The most likely reason for this is the difficulty in the evolution of the appropriate matrix ${\textbf{M}}^{IM}$. In all modules presented in Fig. 13, the first operation represents a linear model. Despite this, none of the modules is ultimately linear. This means that HCMAE was unable to produce the appropriate matrix ${\textbf{M}}^{IM}$ and more operations were required to obtain an accurate model.

**Figure 14:**
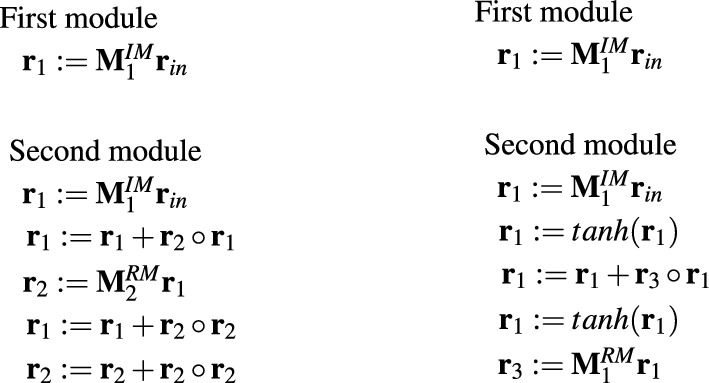
Example effective MOP6 models.

In turn, HCMAE difficulties were most likely due to the placement of matrices ${\textbf{M}}^{IM}$ and ${\textbf{M}}^{RM}$, used by two separate modules, in one common super-matrix and the evolution of this matrix as a separate consolidated module. To obtain two separate linear modules, it would be necessary to create them in two separate locations in the super-matrix. As it turned out, it was easier to expand the MOP modules with further operations than to obtain such a super-matrix.

MOP6 with sequential modular architecture turned out to be the least effective variant of LMGP. It seems that the most reasonable explanation for such a result obtained by MOP6 is the fact that the construction of a reliable model consisting of a sequence of simple operations used by the current version of the LMGP is not an easy task. The linear model is simply the most natural solution in the case considered in the paper, as shown by the tests with monolithic LMGP variants. As already mentioned, most of the models generated by these variants were simple linear models encoded in the form of MOPs consisting of a single operation. More complex models were very rare. Meanwhile, in MOP6, LMGP was forced to construct models composed of a sequence of operations. Of course, an additional difficulty was the same factor as in MOP5, i.e. the use of one super-matrix to represent all the matrices applied in both MOP6 modules.

The construction of reliable models following the MOP6 architecture (see Fig. 14) seems to be the confirmation of the above considerations. Each of these models has a simple linear architecture of an input module and a more complex architecture of an output module. The output module cannot be neutral and pass the input signal without processing which would be equivalent to the solutions obtained for the monolithic LMGP variants. It has to somehow transform the output of the linear module into the final output of the entire model, and the complex architecture of this module, and generally, the results of MOP6, show that this task is a serious challenge for LMGP.

## Limitations

The research reported in the paper shows that LMGP has great potential for effective time series processing, in particular black-box modeling. However, LMGP also has limitations that may hinder its application to more complex problems which may require more complex MOP programs and a larger number of evolving matrices $\mathbf {M^{IM}}$ and $\mathbf {M^{RM}}$.

LMGP uses HCMAE as an optimization engine. One iteration of HCMAE modifies only one matrix among the entire set of matrices. The choice of matrix to change depends on how previous changes to this matrix affected the MOP result. If there are many of these matrices, the process of constructing MOPs may require many iterations of the algorithm.

However, a solution to the above problem may be to use one shared matrix, say $\textbf{SM}$, to encode all the matrices required by LMGP, except the matrix encoding MOP (${\textbf{M}}^{MOP}$). In this case, the task of HCMEA will be to construct two matrices, i.e. one matrix with the list of operations (${\textbf{M}}^{MOP}$) and the second large matrix coding all other matrices used in MOP operations ($\textbf{SM}$). An interesting issue in this case may be the possibility of sharing information among many different matrices used by different MOP operations, but encoded in one common $\textbf{SM}$ matrix modified by HCMEA. This situation will occur if individual MOP matrices overlap in the matrix modified by HCMEA.

Another problem that may arise when generating very large MOPs is memory consumption. LMGP is a population algorithm, which means that its implementation requires storing in memory not only the population of chromosomes but also the MOPs for each chromosome. Moreover, taking into account the fact that MOPs evolve not in one population but in several populations, the problem of memory consumption may be very serious and require appropriate hardware architecture and LMGP implementation.

## Future works

Further research will focus on three areas:

1. The first is to apply LMGP to other time-series problems and to model real objects.
2. The second area is the search for new solutions to increase the effectiveness of LMGP. For example, an interesting issue is the scalability of LMGP to larger problems. Is it better to use longer programs consisting of simple operations such as those used in the current version of LMGP, or shorter programs consisting of more complex operations? Such operations can take the form of groups of simple operations, for example, a combination of IM and RHT operations into one operation. Neural networks with MOP registers as inputs and outputs can also play the role of operations. In this case, the MOPs would be essentially modular neural networks with inter-module connectivity defined by the MOP operations.
3. Another interesting direction of LMGP development is the application of the algorithm to the evolution of feed-forward layered neural networks like convolutional networks. In this case, each MOP operation should generate input for the next operation in the program, what is more, they should implement actions on the images.

## Summary

The paper presents Linear Matrix Genetic Programming, which is a novel evolutionary algorithm for modeling complex dynamic objects in the form of sequences of simple matrix operations called Matrix Operation Programs. The algorithm was validated on data representing the behavior of an underwater vehicle. The data was collected during tests in simulation conditions aimed at constructing the control system of an underwater vehicle. Various types of neural networks, feed-forward and recurrent, monolithic and modular, evolutionary and trained with gradient algorithms, were used as a reference point for the proposed method.

The experiments reported in the paper revealed that LMGP models are as effective as the most accurate neural models. The maximum accuracy of the models expressed by the function (5), in the case of the LMGP as well as two neuro-evolutionary models, was above 0.38. In turn, the average accuracy of three out of six LMGP models and again two neuro-evolutionary models was above 0.35. For comparison, the accuracy of the most efficient LSTM/GRU models, which are the standard when it comes to black-box neural models, was 0.125 and 0.095, respectively.

However, the advantage of LMGP models is that they are easier to analyze than their neural counterparts. For most of us, analyzing the sequence of matrix operations is much simpler than analyzing nonlinear processing units connected somehow in a network.

Although MOPs are the counterpart of LSTM and GRU recurrent networks with a configurable internal architecture, they proved to be a much more effective modeling tool than the mentioned LSTM/GRU networks. However, the reason for such a state of affairs does not have to be the construction of the LSTM/GRU networks themselves, but the algorithm that was used to train them. MOPs were trained evolutionarily, while to train LSTM/GRU networks, gradient algorithms were used, which tend to get stuck in local minima.

LMGP can be also applied to evolve modular MOPs whose modular architecture is known in advance. In the modeling problem considered in the paper, modular MOPs with parallel organization appeared to be as effective as their monolithic counterparts, whereas sequentially organized MOPs turned out to be the least effective LMGP variant. Although MOPs themselves are a sequence of operations and monolithic variants generated reliable sequential models, the imposed sequential modular MOP architecture in which two modules have to fit perfectly together turned out to be more difficult to implement than a solution in which LMGP had the freedom to shape models.

## Parameters of HCMAE applied in the tests

The following parameters of HCMAE were applied in the tests: the number of populations = 3 (one population with data and two populations with operations), size of each population = 100, length of chromosome—data = 50 real-valued genes, length of chromosome—operations: 6 integer genes, crossover probability in each population = 0.5, $P^{d}_{m}=0.1, P^{o}_{m}=0.8$ (mutation probabilities), $P^{o, zero}_{m}=0.3$ (probability of a mutated gene to be zero), range of mutation $a=0.4$, $b=3$ (see Eq. 8 and 9), size of tournament=3, $MAX\_HOLES=3$ (the maximum size of contiguous patches in the matrix that were cleared by operations). HCMAE was aborted after 400 iterations of the while loop (see Algorithm 1)

The chromosomes—data and chromosomes—operations were mutated differently, and it was performed as follows:

8 $$\begin{aligned} d_{new}= & {} {\left\{ \begin{array}{ll} d + \textrm{randU}(-a,a)\; \textrm{if}\; \textrm{randU}(0,1)\le P^{d}_{m}\\ d\;\textrm{otherwise} \end{array}\right. } \end{aligned}$$

9 $$\begin{aligned} o_{new}= & {} {\left\{ \begin{array}{ll} o + \textrm{randI}(-b,b)\; \textrm{if}\; \textrm{randU}(0,1)\le P^{o}_{m}\; \textrm{and} \; \textrm{randU}(0,1)\ge P^{o, zero}_{m}\\ 0 \; \textrm{if}\; \textrm{randU}(0,1)\le P^{o}_{m}\; \textrm{and} \; \textrm{randU}(0,1)\le P^{o, zero}_{m}\\ o\;\textrm{otherwise} \end{array}\right. } \end{aligned}$$

where

- *d*—is a gene in a chromosome—data
- *o*—is a gene in a chromosome—operation
- randU($-a,a$)—is a uniformly distributed random real value from the range $<-a,a>$
- randI($-b,b$)—is a uniformly distributed random integer value from the range $<-b,b>$.

## Data Availability

The dataset used during the research will be made available upon request. To obtain the datasets, please contact the first author of the paper: t.praczyk@amw.gdynia.pl.
